# System for the Unified Management, Assessment, and Review of Information (SUMARI)

**DOI:** 10.5195/jmla.2019.790

**Published:** 2019-10-01

**Authors:** Christi Piper

**Affiliations:** Reference Librarian, Strauss Health Sciences Library, University of Colorado Anschutz Medical Campus, Aurora, CO, christi.piper@ucdenver.edu

## INTRODUCTION

Due to the overwhelming number of studies published each year, systematic reviews are becoming increasingly important in health care settings. Systematic reviews aim to provide a comprehensive, unbiased summary of all of the relevant studies for a specific health care issue, emphasizing specifically the use of rigorous methods that are reproducible. Librarians support the development of systematic reviews in a variety of ways, including but not limited to, serving as expert searchers, methodologists, protocol development assistants, and other roles.

In an attempt to develop rigorous procedures and practices for systematic reviews, a number of organizations, such as Cochrane and the Joanna Briggs Institute (JBI), have created guidelines and manuals for the process. [1, 2] These manuals provide guidance on all of the steps involved in a systematic review including search development and best practices for review procedures, data extraction, and data analysis, all with the goal of reducing the risk of error and bias in the review process. In addition to these manuals, standards are available to improve the reporting of systematic reviews, such as the Preferred Reporting Items for Systematic Reviews and Meta-Analyses (PRISMA), that provide a minimum set of required items. With the guidance of one such manual, JBI created the System for the Unified Management, Assessment, and Review of Information (SUMARI) to walk researchers through the entire systematic review process.

## OVERVIEW

SUMARI is a web-based review application that supports the majority of steps involved in the review process. There are multiple ways that an individual can obtain access to SUMARI: attend the JBI Comprehensive Systematic Review Training Program to receive free access for 12 months; utilize an institutional subscription to any JBI resource provided by the Wolters Kluwer Ovid platform where SUMARI can be found in the evidence-based practice (EBP) Tools menu for no additional cost (an Ovid personal account is required); or purchase an individual subscription through Lippincott’s Nursing Center on the Evidence-Based Practice Network for $130 annually.

SUMARI is based on the JBI Reviewer’s Manual and provides functionality to align with the JBI process and requirements. Researchers can choose from ten different review types or create their own custom review. Once the review type has been selected, a new project is created. The project landing page is the Overview tab with a Summary screen and a Participants screen. The Summary screen displays information about the numbers of studies left to review, appraise, and extract data from, while the Participants screen allows other researchers to be added to the project.

Participants can be added to one of four roles: Project Owner, Privileged Author, Author, or Reviewer. The Project Owner has the highest level of permission, and the Reviewer has the lowest. Each participant must have their own subscription access to SUMARI to access the project. All the content created in SUMARI can be exported to a Word document in the individual sections.

## PROGRESSING THROUGH THE REVIEW

**Figure 1:**
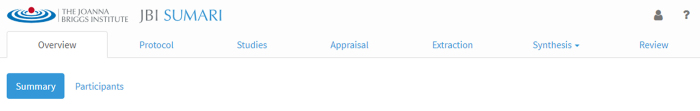
SUMARI progress tabs

Tabs at the top of the web page guide researchers through the entire review process from Protocol to Synthesis (Figure 1).

### Protocol

When a project is created, the researcher selects a review type. The selected review type corresponds to the Protocol template provided in this section. The template supplies descriptions of the content that should be included in each section of the protocol, for example, the abstract, introduction, and methods sections. Highlighting in the template is used to indicate content that researchers are required to insert for their specific reviews.

### Studies

The Studies section allows search results to be imported via an XML or RIS file, or via manual citation creation. Once studies are uploaded, the title and publication year of each is displayed for inclusion and exclusion review. Reasons for exclusion must be provided. Previously used reasons are populated in a dropdown menu as they are created. Included studies move to the Appraisal section.

### Appraisal

The Appraisal section is used for included studies via thirteen different study design–based appraisal forms and the Cochrane Risk of Bias tool. The critical appraisal tools are the standardized tools that are required for use in JBI reviews. The default for critical appraisal is two reviewers but can be overridden to allow one individual reviewer. When both reviewers have completed independent critical appraisals, a third critical appraisal form displays the alignment of both reviewers’ responses and creates a final appraisal for the selected study. Whether or not the appraisal is completed, studies are available in the Extraction section.

### Extraction

The Extraction section allows researchers to begin pulling out critical data from studies to prepare for synthesis. Once an extraction record is created for a study, only the record creator or review owner is allowed to edit it. The Extraction record provides recommended data fields for the researcher to complete based on the critical appraisal form used for individual studies.

### Synthesis

The Synthesis section assists in creating qualitative findings and meta-analysis. The Qualitative option allows researchers to input findings for each of the studies included from the final review and to rank the credibility of findings. Once findings are entered for all studies, the process allows researchers to create categories of findings, provide a description of each, and assign findings to the appropriate category. Once all findings are assigned, the categories can be synthesized through assigning categories to descriptive statements, which then allows a flowchart of the findings to be created. Alternatively, the Meta-Analysis section creates forest plots for outcomes. To create the forest plot, researchers must input the parameters of the meta-analysis; select the statistical method, effect measure, and confidence internal; and then enter the appropriate study data.

### Review

The Review section starts a new review with the current protocol and its associated citations.

## ADVANTAGES AND DISADVANTAGES

The SUMARI interface has a pleasing user-friendly design that provides researchers with a quick-look overview of the review progress along with easy-to-identify action buttons and tasks. The software provides assistance with every part of the review process, and quick tutorial videos are available from the Help menu to demonstrate the basics of using the interface. Additionally, since it is an online platform, there is no software to install.

While the overall design and functionality of SUMARI is well done, there are some aspects of the software that researchers will want to be aware of. For study upload, there is no de-duplication function in SUMARI, and no warning is provided if duplicates are present. Researchers will want to download search results into a citation management program such as EndNote from Clarivate to de-duplicate results before uploading them into SUMARI. Once studies are uploaded, SUMARI only allows a final review and does not provide the option to upload the full text of studies. JBI recommends utilizing Covidence (reviewed in the October 2018 issue of the *Journal of the Medical Library Association*) for the screening process and then uploading the final studies into SUMARI to continue the review process. While this partnership is mentioned, it is assumed that researchers will need to purchase access to Covidence because it is not provided with SUMARI. Finally, SUMARI does not provide a PRISMA diagram at the conclusion of a review, so that needs to be managed separately by the researcher.

## CONCLUSION

JBI’s SUMARI is easy-to-use software that walks researchers through the entire review process from start to finish. Integrated with the JBI Reviewer’s Manual, SUMARI provides an easy-to-understand method for the review process that would be beneficial for those who are new to systematic reviews. The software is comprehensive enough that the shortcomings of the program can be manageable if other software applications such as EndNote and Covidence are available to the researcher. Librarians may find the basic help and support information that is provided lacking, but researchers will appreciate the comprehensive guide to the review process.

## 

***Christi Piper****, christi.piper@ucdenver.edu, Reference Librarian, Strauss Health Sciences Library, University of Colorado Anschutz Medical Campus, Aurora, CO*
